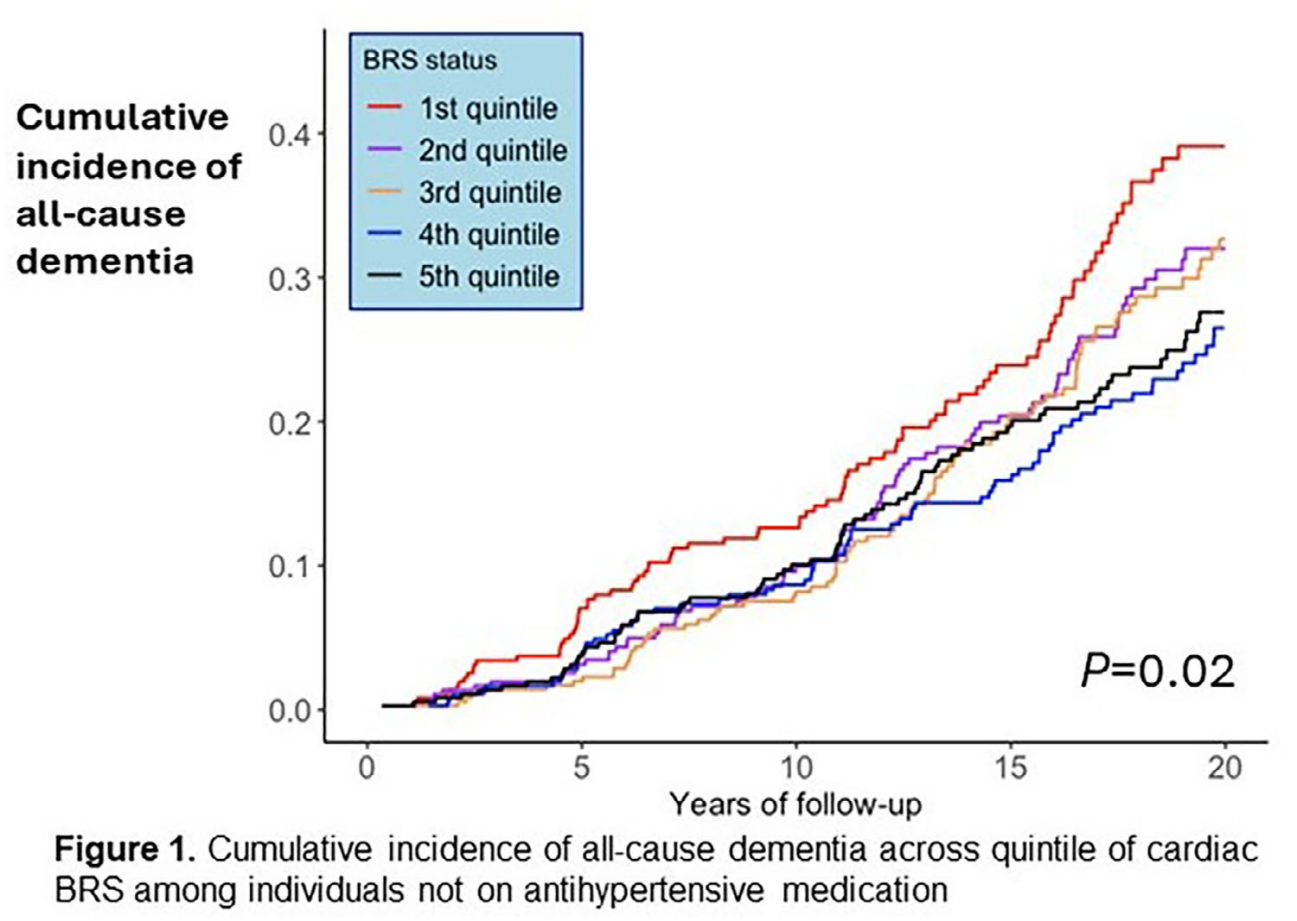# Impaired baroreflex sensitivity and long‐term risk of dementia in community‐based older adults

**DOI:** 10.1002/alz.088038

**Published:** 2025-01-09

**Authors:** Yuan Ma, Yiwen Zhang, Rikuta Hamaya, Berend E Westerhof, Hossam A Shaltout, Maryam Kavousi, Francesco U S Mattace Raso, Albert Hofman, Frank J. Wolters, Lewis A Lipsitz, M. Arfan Ikram

**Affiliations:** ^1^ Harvard T.H. Chan School of Public Health, Boston, MA USA; ^2^ Harvard T. H. Chan School of Public Health, Boston, MA USA; ^3^ Brigham and Women’s Hospital, Boston, MA USA; ^4^ Amsterdam UMC, Vrije Universiteit Amsterdam, Amsterdam Netherlands; ^5^ Westerhof Cardiovascular Research, Amsterdam Netherlands; ^6^ Wake Forest University School of Medicine, Winston‐Salem, NC USA; ^7^ Department of Epidemiology, Erasmus MC, Rotterdam, Netherlands, Rotterdam Netherlands; ^8^ Erasmus MC University Medical Center, Rotterdam Netherlands; ^9^ Erasmus University Medical Center, Rotterdam, Zuid‐Holland Netherlands; ^10^ Hebrew SeniorLife Hinda and Arthur Marcus Institute for Aging Research, Beth Israel Deaconess Medical Center, and Harvard Medical School, Boston, MA USA

## Abstract

**Background:**

Hypertension is an important modifiable risk factor for dementia, but the role of blood pressure (BP) in the development of dementia is not fully understood. Emerging data links increased BP variability and abnormal BP dynamics to dementia risk, but the relationship between baroreflex sensitivity (BRS), a fundamental physiological mechanism for maintaining stable BP, and dementia risk is unknown.

**Methods:**

We investigated the association of BRS with the risk of dementia in community‐based older adults from the Rotterdam Study in the Netherlands. We determined cardiac BRS using a 5‐minute continuous beat‐to‐beat recording of BP and heart rate measured in a supine position between 1997 and 1999 and ascertained incident dementia cases using a standardized protocol from baseline through January 1, 2020. We quantified the association of cardiac BRS with dementia risk using adjusted cause‐specific hazard ratios.

**Results:**

Of 1,819 participants (63% women; mean [SD] age, 71.0 [6.3] years), 421 developed dementia during a median follow‐up of 14.8 years. The association of cardiac BRS with dementia risk differed by antihypertensive medication use (*P* for interaction = 0.03) and was only observed in participants not taking antihypertensive medication. Specifically, reduced BRS was associated with a higher risk of dementia (cause‐specific hazard ratio [HR] comparing bottom *versus* top quintiles: 1.62; 95% confidence interval [CI]: 1.08‐2.42, *P* for trend = 0.02) and all‐cause mortality (corresponding HR: 1.77; 95%CI: 1.33‐2.37, *P* for trend<0.001) after adjusting for age, sex, *APOE* genotype, and traditional vascular risk factors. The association remained after additional adjustment for sitting BP level and beat‐to‐beat BP variability.

**Conclusions:**

Among older adults not taking antihypertensive medication, reduced cardiac BRS was associated with a higher long‐term risk of dementia and all‐cause death over 23 years. Impaired BRS may be a novel biomarker of significant clinical relevance for early detection and prevention of dementia in older adults.